# Severe dengue in adults: Clinical features from the 2022 dengue outbreak at a Vietnamese tertiary hospital

**DOI:** 10.1371/journal.pntd.0013589

**Published:** 2025-10-03

**Authors:** Oanh Kim Pham, Thuy Bich Duong, Tho Vinh Phan, Trung Ngoc Truong, Duong Thi Hai Ha, Hao Van Nguyen, Vu Thi Minh Hien, Trinh Huu Khanh Dong, Nguyen Lam Vuong, Lam Minh Yen, Sophie Yacoub, Catherine Louise Thwaites, Dong Thi Hoai Tam

**Affiliations:** 1 Department of Infectious Diseases, University of Medicine and Pharmacy at Ho Chi Minh City, Ho Chi Minh City, Vietnam; 2 Infections Diseases Department, FV Hospital, Ho Chi Minh City, Vietnam; 3 Department D, Hospital for Tropical Diseases, Ho Chi Minh City, Vietnam; 4 Adult Intensive Care Unit, Hospital for Tropical Diseases, Ho Chi Minh City, Vietnam; 5 Emerging Infections Group, Oxford University Clinical Research Unit, Ho Chi Minh City, Vietnam; 6 Biostatistics Group, Oxford University Clinical Research Unit, Ho Chi Minh City, Vietnam; 7 Faculty of Public Health, University of Medicine and Pharmacy at Ho Chi Minh City, Ho Chi Minh City, Vietnam; 8 Dengue Group, Oxford University Clinical Research Unit, Ho Chi Minh City, Vietnam; 9 Nuffield Department of Medicine, University of Oxford, Oxford, United Kingdom; Public Health Agency of Canada, CANADA

## Abstract

**Background:**

In Vietnam, dengue has been endemic for many years, with most cases reported in children. Recently, epidemiological data show an increasing frequency in adults, especially for severe dengue. An unprecedented post-COVID-19 surge resulted in an exceptionally high number of hospitalized dengue cases. We aim to describe the clinical phenotypes and outcomes in Vietnamese adults with severe dengue during the 2022 outbreak and explore host-related factors associated with disease variability and severity, through a retrospective study.

**Findings:**

A total of 891 cases were included, with mean age 29 ± 10 years. 284/891 (31.9%) patients had a BMI ≥ 25 kg/m^2^, and 240/891 (26.9%) had comorbidities. The predominant severe clinical phenotype was dengue shock syndrome (DSS): 737/891 (82.7%) patients. 107/891 (12%) DSS cases were associated with other severe manifestations. Severe hemorrhage accounted for 90/891 (10.1%) patients. Among cases with organ involvement (211/891 - 23.7%), hepatic impairment was observed in 196/891 (22%) patients, renal impairment 25/891 (2.8%), cardiac impairment 14/891 (1.6%) and neurological impairment 13/891 (1.5%). 250/737 (33.9%) DSS patients developing ≥ 1 episode of recurrent shock. They were younger than those without recurrent shock (25.3 vs 28.4 years, p = 0.007). Factors associated with recurrent shock episodes were: having BMI ≥ 25 (OR: 1.65; 95% CI: 1.18; 2.3), day of illness ≤ 5 (OR: 2.16; 95% CI: 1.51; 3.09) and prior COVID-19 infection (OR: 2.57; 95% CI: 1.62-4.06). Indicators for the “associated severe phenotypes” (DSS associated with severe hemorrhage, with organ impairment, or both) were older age (p = 0.018) and presence of comorbidities (p < 0.001) compared to the DSS alone phenotype. Overall, 98.1% of patients had a good recovery.

**Conclusions:**

Understanding the variability and complexity of severe dengue clinical manifestations, along with the different host factors associated with these features, will contribute to formulating suitable treatment guidelines for this at-risk population.

## Author summary

The incidence of dengue infection has increased over the past two decades. Especially after the COVID-19 pandemic, the number of cases has increased significantly, according to World Health Organization reports. In 2024, there were 90 countries that had known active dengue transmission. Currently, there is still no specific treatment for the disease. Prevention measures mainly rely on vector control. Vaccines have been introduced, but their effectiveness remains limited. Many dengue cases are mild; however, some cases will develop severe dengue, which may involve shock, severe hemorrhage or organ impairment. According to many reports, the rate of dengue fever in adults is increasing compared to children. This may be due to improved environmental sanitation, a more developed healthcare system that enables better disease diagnosis, and longer human life expectancy. Previous studies have often focused on describing dengue in children. Adults, with fully developed body structures, different body fluid distribution compared to children, and the possibility of having chronic illnesses, may require different approaches in treatment and in identifying risk factors for severe disease. Therefore, researching adult patients with dengue fever will provide clinicians with more knowledge about the disease, aiding in monitoring and detecting its severe progression.

## Introduction

Dengue is a common mosquito-borne viral infection in tropical climates and a major health burden in many countries. Worldwide, reported cases increased from 500,000 in 2000 to 5.2 million in 2019, characterized by the simultaneous occurrence of multiple outbreaks [1]. In 2023, an upsurge in dengue cases was observed globally [2,3]. According to World Health Organization (WHO) 2009 guidelines, dengue infection is classified as dengue without warning signs, dengue with warning signs and severe dengue. Severe dengue may be accompanied by shock or other features such as disseminated intravascular coagulation, liver failure, renal failure, acute respiratory failure and patients may die if not promptly detected and treated [4].

In Vietnam, dengue has been endemic for many years, but its epidemiology is changing; although historically affecting children, the disease now affects more adults than children [5]. Concurrently with this is the rising incidence of severe dengue, from 11% in 2011–2017 [6,7] to 17.7% in 2022 - incidence observed from 11,412 hospitalized adult patients at the Hospital for Tropical Diseases (HTD), a tertiary referral infectious diseases hospital for southern Vietnam (annual report from the Planning Department of HTD). Data from WHO shows an increase in mortality due to dengue between 2020 and 2022 in Vietnam [8,9].

In the context of this changing epidemiology and burden of disease, there is uncertainty about the manifestations and evolution of severe disease in adults. To address this knowledge gap, we have carried out a detailed description of adults hospitalized with severe dengue, to better understand the drivers of these severities and to better inform therapeutic options and research.

## Methods

### Ethics statement

The study was approved by the Ethical Committee of the Hospital for Tropical Diseases and the Oxford Tropical Research Ethics Committee. As it was a retrospective study, there was no written informed consent obtained from the participants, whose identification were obtained through the ICD (International Classification of Diseases) codes determined at discharge.

### Study design and population

This was a retrospective study, conducted at the HTD, Ho Chi Minh city (HCMC). We reviewed relevant medical records of hospitalized patients at HTD, from 1^st^ June 2022–31^st^ December 2022, corresponding to the rainy season in southern Vietnam and peak dengue season.

Patients’ demographic characteristics, history, clinical manifestations and laboratory parameters at the time for inclusion in the study and during hospitalization were collected from the hospital files to an electronic system using a specially designed case record form.

Patients were included in the analysis if they were aged ≥ 16 years old, had disease onset ≤ 7 days, diagnosed as severe dengue, based on the standard definitions as per pre-existing 2009 WHO Guidelines for dengue diagnosis, treatment, prevention and control [4]. Severe dengue was defined by one or more of the following clinical manifestations: shock and/or evidence of severe plasma leakage or fluid accumulation, severe hemorrhage, organ impairment (liver injury, kidney injury, cardiomyopathy, encephalopathy). Confirmed dengue cases were based on either a positive NS1 antigen test or an IgM ELISA performed in the hospital laboratory. However, with patients admitted late in the disease course (more than day 5–6 of illness), clinicians sometimes opted not to perform NS1 testing due to the low sensitivity of the test in the late febrile phase. The clinical diagnosis for these patients is rather based on a combination of epidemiological information, symptoms, disease progression, and laboratory parameters, including daily changes in blood counts, transaminase elevation, and/or evidence of pleural effusion or ascites. Therefore, we also included patients without these 2 tests if the clinical symptoms and evolution during hospitalization were consistent with dengue disease and after excluding other diagnoses. A summary of the different study definitions for severe dengue phenotypes was presented in the Appendix (S1 Appendix).

### Statistical analysis

Study data were entered into a dedicated secure database and SPSS 26.0 software was used for analysis. Continuous variables were described using mean and standard deviation or median and interquartile range (IQR). Categorical variables were described using the number of patients and the percentage. Differences between two groups (Dengue shock syndrome (DSS) with and without recurrent shock) were tested using two-sample t-test or Mann-Whitney-U test for continuous variables, and Chi-squared test or Fisher’s exact test for categorical variables. Differences between four groups (DSS alone, DSS + severe hemorrhage, DSS + organ impairment, and DSS + organ impairment + severe hemorrhage) were tested using one-way ANOVA test for continuous variables and Chi-squared test or Fisher’s exact test for categorical variables. In case of significant difference, a post-hoc ANOVA test was used to compare each paired group. All tests were two-sided and p-value < 0.05 was considered statistically significant.

## Results

### Baseline characteristics

From June to December 2022, a total of 8,951 patients diagnosed with dengue infection were admitted to HTD, identified at discharge according to ICD codes corresponding to the severity of dengue. Among these, 891 patients met our study inclusion criteria. Their demographic characteristics are summarized in Table 1. The proportion of male and female patients was similar (46.9% vs 53.1%). The mean age of patients was 29 ± 10 years. Only 8 patients (0.9%) were aged over 60 years, the oldest being 76 years old. Patients with BMI ≥ 25 accounted for nearly one-third of the population: 284 cases (31.9%), while there were only 66 cases (7.4%) with BMI < 18. There were 15 pregnant women out of 473 females (3.2%). 240 (26.9%) patients reported having comorbidities, with liver disease being the most common (155 patients, 17.4%). The list of comorbidities is presented in Table 2.

**Table 1:** Baseline demographic characteristics and outcome of the study population (N = 891).

Characteristics	Summary statistics (N = 891)
**Gender**	
Male	418 (46.9)
Female	473 (53.1)
**Age (years)**	29 ± 10
**Age groups**	
16-20	229 (25.7)
21-40	538 (60.4)
41-60	117 (13.4)
> 60	8 (0.9)
**BMI** **≥ 25 kg/m**^**2**^	284 (31.9)
**Pregnancy n (%) N = 473**	15 (3.2)*
**Having comorbidities**	240 (26.9)
Liver disease	155 (17.4)
Hypertension	32 (3.6)
Diabetes	30 (3.4)
**Past history of COVID-19 infection**	109 (12.2)
**Living areas**	
Ho Chi Minh city	524 (58.8)
Other provinces	367 (41.2)
**Transfer from other health settings**	424 (57.6)
**Median Day of illness at enrollment** (days)	5 (5; 6)
**Confirmed by positive NS1 or IgM *Dengue***	475 (53.3)
**Length of stay in hospital** (days)	5 (4; 6)
**Patients admitted to ICU**	199 (22.3)
**Length of stay in ICU** (days)	2 (2; 4)
**Complications (hospital-acquired infection)**	67 (7.5)
**Discharge status**	
Good recovery	874 (98.1)
Death	11 (1.2)
Recover with sequelae	3 (0.3)
Transfer to another hospital	3 (0.3)**

*For 15 pregnant women: 6 were in first trimester, 4 in 2^nd^ trimester and 5 in 3^rd^ trimester.

**Transfer due complications (1/3 needed hemodialysis for chronic kidney failure, 2/3 were miscarriage).

Summary statistics are n (%), median (IQR) or mean ± SD.

COVID-19: Coronavirus disease 2019; NS1: Non-structural protein 1; ICU: Intensive Care Unit.

**Table 2:** Most common comorbidities (N = 891).

Characteristics	Summary statistics (N = 891)
Liver disease	155 (17.4)
Fatty liver disease	136 (15.3)
Alcoholic hepatitis	16 (1.8)
Chronic hepatitis B	3 (0.3)
Hypertension	32 (3.6)
Diabetes	30 (3.4)
Peptic ulcer	18 (2)
Hematologic disease	8 (0.9)
G6PD deficiency	2 (0.2)
Thalassemia	1 (0.1)
Anemia	4 (0.4)
Idiopathic thrombocytopenia	1 (0.1)
Cardiac disease	9 (1)
Cardiac arrhythmia	2 (0.2)
Valvular heart disease	5 (0.6)
Myocardial infarction	2 (0.2)
Renal disease	13 (1.5)
Nephrotic syndrome	7 (0.8)
Lupus	2 (0.2)
Acute glomerulonephritis	1 (0.1)
Chronic kidney disease	3 (0.3)
Any pulmonary disease	6 (0.7)
Cancer of any type	4 (0.4)
Epilepsy	6 (0.7)
Dementia	4 (0.4)
Growth retardation	3 (0.3)
Hyperthyroidism	2 (0.2)
Hypothyroidism	2 (0.2)
Anxiety disorders	1 (0.1)
Arthritis - prolonged use of anti-inflammatory drugs	1 (0.1)

Summary statistics are n (%).

424 patients (57.6%) were transferred from other districts or provincial hospitals, and 77.8% of them (330/424) had stayed in the initial hospital less than 48 hours. The median illness day at inclusion in the study was Day 5 (IQR: 5;6), aligning with the period when severe dengue manifestations typically occur. Notably, 4 patients presented on Day 1–2 of illness with neurological symptoms, and 3 patients presented on Day 2 with severe bleeding signs and significant liver impairment.

### Clinical phenotypes of severe dengue

**Fig 1:**
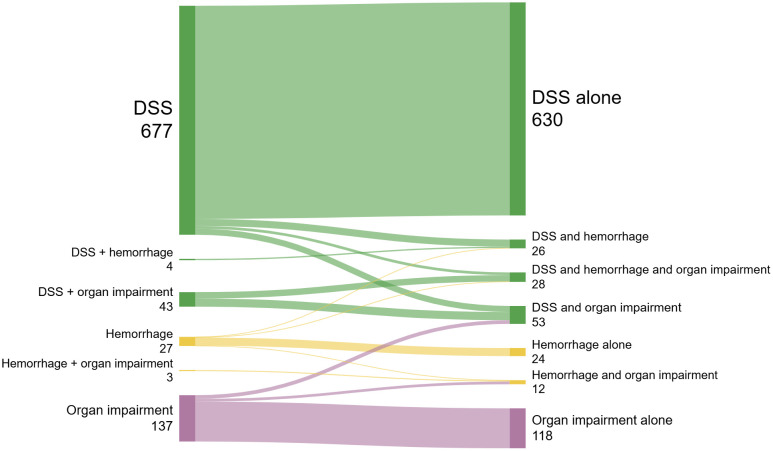
Progress of clinical phenotypes from enrollment to discharge (N = 891). DSS: Dengue shock syndrome.

We classified the different clinical phenotypes collected at 2 time points: on the day of enrollment (inclusion day in the study) and the day of discharge (Table 3), explaining the dynamic progression of the disease during illness (illustrated by Fig 1). For most patients DSS alone was the initial feature (677 patients - 76%), compared to severe hemorrhage alone (3%) or organ impairment alone (15.4%). Evolution during hospitalisation was inconsistent, with some patients presenting with multiple features and others with a more stepwise involvement of organs. There were no cases with concomitant shock, hemorrhage and organ impairment at enrollment, however this feature constellation was observed in 28 patients (3.1%) at discharge.

**Table 3:** Clinical phenotypes of severe dengue at enrollment and discharge (N = 891).

	AT ENROLLMENT		AT DISCHARGE*	
	One phenotype n (%)	Associated phenotypes n (%)	One phenotype n (%)	Associated phenotypes n (%)
**Dengue Shock Syndrome (DSS)**	677 (76)	724 (81.3)	630 (70.7)	737 (82.7)
DSS + hemorrhage		4 (0.4)		26 (2.9)
DSS + organ impairment		43 (4.8)		53 (5.9)
DSS + hemorrhage + organ impairment		0		28 (3.1)
**Hemorrhage**	27 (3.0)	34 (3.8)	24 (2.7)	90 (10.1)
Hemorrhage + organ impairment		3 (0.3)		12 (1.3)
**Organ impairment**	137 (15.4)	183 (20.5)	118 (13.2)	211 (23.*7*)
Hepatic impairment	124 (13.9)		105 (11.8)	196 (22)
Renal impairment	0 (0)		0 (0)	25 (2.8)
Cardiac impairment	2 (0.2)		2 (0.2)	14 (1.6)
Neurological impairment	9 (1)		6 (0.7)	13 (1.5)
Multi-organ impairment	2 (0.2)		5 (0.5)	

*: the most severe form of dengue during hospitalization, either alive or dead.

One phenotype: The patient has only one severe form of dengue infection (e.g., dengue shock syndrome (alone) or hemorrhage (alone) or organ impairment (alone). Associated phenotypes: The patient may have one or multiple severe forms.

We classified the different clinical phenotypes collected at 2 time points: on the day of enrollment (inclusion day in the study) and the day of discharge (Table 3), explaining the dynamic progression of the disease during illness (illustrated by Fig 1). At enrollment, 124 of 891 patients (13.9%) presented with hepatic impairment, of whom 28 (22.6%) had pre-existing liver disease as a comorbidity.

The three most common of severe dengue were dengue shock syndrome (737/891 patients, 82.7%), hepatic impairment (196/891 patients, 22%), and severe hemorrhage (90/891 patients, 10.1%). A summary description of these 3 phenotypes was presented in Table 4.

**Table 4:** Description of dengue shock syndrome, severe hemorrhage and hepatic impairment phenotypes.

Characteristics	N	Summary statistics
**Dengue shock syndrome N = 737**		
Day of illness when developing shock (day)	737	5 (4; 6)
Patients having recurrent episodes	737	250 (33.9)
1 episode		133 (17.6)
2 episodes		92 (12.5)
≥ 3 episodes		25 (3.4)
Haematocrit at shock (%)	715	51.8 ± 5.5
Platelets at shock (K/µl)	737	15 (9; 24)
**Fluid therapy for Shock treatment**		
Colloids	737	313 (42.5)
Albumin	737	114 (15.5)
Total period for fluid perfusion (hours)	737	24 (24; 32)
Total volume of fluid (ml/kg)	737	86 (86; 120)
Total volume of colloid (ml/kg)	737	20 (15; 35)
**Oxygen support**		
HFNC (high flow nasal cannula)	160	34 (21.3)
NIV (noninvasive ventilation)	160	18 (11,3)
Mechanical ventilation	160	24 (15)
**Dengue with severe hemorrhage (n = 90)**		
**Day of illness with severe hemorrhage**	90	5 (5; 6)
**Hemorrhage site**		
Nose	90	24 (26.7)
Mouth	90	15 (16.7)
Upper digestive tract	90	43 (47.8)
Venipuncture site	90	23 (25.6)
Vagina	47	19 (40.4)
More than 1 site	90	41 (45.6)
**Platelets (K/µL)**	90	13 (6.8; 28)
**INR**	83	1.2 (1.1; 1.8)
**Fibrinogen (g/l)**	82	2 (1.4; 2.7)
**Blood product for bleeding management**		
Fresh-packed red cells	90	56 (62.2)
Frozen plasma	90	34 (37.8)
Cryoprecipitate	90	15 (16.7)
Platelets	90	68 (75.6)
**Intervention in situ**		
Nose packaging	90	22 (24.4)
Gastric endoscopy*	90	3 (3.3)
Local compression	90	9 (10)
**Hepatic impairment (n = 196)**		
**Day of illness**	196	5 (5; 6)
**Jaundice**	196	59 (30.1)
**Acute liver failure**	196	58 (29.6)
**Encephalopathy**	196	11 (5.6)
**AST max (U/L)**	196	2 004 (1 335; 3 639)
**ALT max (U/L)**	196	882 (647; 1 544)
**Bilirubin max (µmol/l)**	170	23.8 (13; 47.9)
**INR max**	178	1.2 (1; 1.6)
**Treatment**		
Use N- Acetylcystein	196	9 (4.6)
Use Therapeutic plasma exchange	196	7 (3.6)

*1 case: Forrest IB, 2 cases: Forrest IIC.

Summary statistics are n (%), median (IQR) or mean ± SD.

INR: international normalized ratio: AST: aspartate aminotransferase; ALT: alanine aminotransferase.

**Dengue shock syndrome:** This most important feature of severe dengue disease, occurring alone or in association with severe hemorrhage or organ impairment. 250/737 (33.9%) patients had ≥ 1 episode of recurrent shock during 24–48 hours of initial crystalloid perfusion resuscitation. Per management guideline, patients having severe plasma leakage (resulting in many recurrent shock episodes) received colloids or albumin, associated with signs of ascites, pleural effusion and respiratory distress (160/737 cases, 21.7%). Mechanical ventilation was required in 24 of these 160 cases (15%).

**Severe hemorrhage:** The most common bleeding site was the upper gastrointestinal tract (43 cases, 47.8%). We noticed a high percentage of vaginal bleeding in female patients (19/47 cases -40.4%) and almost half of the patients had more than 1 site of bleeding. Management of severe thrombocytopenia or coagulation system disturbance resulted in a high percentage of blood product transfusions, and fewer cases required in situ intervention.

**Liver impairment:** 58 cases in these 196 patients (29.6%) developed acute liver failure, 59 cases (30.1%) had jaundice and 11 cases were diagnosed with encephalopathy (5.6*%).* Liver enzyme values were very high with a median AST maximum of 2004 U/L (IQR: 1335; 3639). We also found 8 cases with severe hepatic impairment leading to severe hemorrhage 1 or 2 days later due to coagulation disorders.

### Factors associated with severe disease progression

Severe disease progression was defined as an evolution involving a more severe feature, compared to the feature at first presentation.

**Recurrent shock:** Many episodes of recurrent shock observed in DSS patients were a complicated evolution leading to more difficulties in the management such as admission to ICU, additional infusion of colloids or albumin resulting in pleural effusion, ascites and respiratory distress features. The two groups of patients with DSS with and without recurrent shock are shown in Table 5. We excluded patients who had simultaneously shock and severe hemorrhage, avoiding recurrent shock status due to hemorrhage. Among these 683 patients, those with recurrent shock were younger than those without (25.3 vs 28.4 years, p = 0.007). We also found that recurrent shock was associated with patients with BMI ≥ 25 (OR: 1.65; 95%CI: 1.18; 2.3; p = 0.003), with patients having prior COVID 19 infection (OR: 2.57; 95%CI: 1.62-4.06; p < 0.001), and day of illness at shock presentation ≤ 5 (OR: 2.16; 95% CI: 1.51; 3.09; p < 0.001).

**Table 5:** Factors associated with recurrent episodes among patients having dengue shock syndrome (N = 683).

Risk factor	DSS without recurrent shock N = 466	DSS with recurrent shock N = 217	OR (95%CI)	P value
**Age**	28.37 ± 9.16	26.33 ± 9.39		0.007**
**Age group**				
16-20	113 (59.2)	78 (40.8)	1.69 (0.95-3.01)	0.070
21-40	299 (71.9)	117 (28.1)	0.96 (0.56-1.65)	0.890*
> 40	54 (71.1)	22 (28.9)	Ref	
**Gender**				
Male	241 (66.6)	121 (32.4)	Ref	
Female	225 (70.1)	96 (29.9)	0.85 (0.62-1.18)	0.324
**BMI**				
< 25	322 (72.0)	125 (28.0)	Ref	
≥ 25	144 (61.0)	92 (39.0)	**1.65** (**1.18-2.3)**	**0.003**
**Having comorbidities**				
No	366 (69.7%)	159 (30.3%)	Ref	
Yes	100 (63.3%)	58 (36.7%)	1.36 (0.92-1.94)	0.128
**Liver disease**				
No	394 (68.9)	178 (31.1)	Ref	
Yes	72 (64.9)	39 (35.1)	1.2 (0.78-1.84)	0.406
**Hypertension**				
No	455 (68.5)	209 (31.5)	Ref	
Yes	11 (57.9)	8 (42.1)	1.58 (0.63-4)	0.327*
**Diabetes**				
No	458 (68.5)	211 (31.5)	Ref	
Yes	8 (57.1)	6 (42.9)	1.63 (0.56-4.75)	0.391*
**History of COVID-19 infection**				
No	424 (71)	173 (29)	Ref	
Yes	42 (48.8)	44 (51.2)	**2.57** (**1.62-4.06)**	**<0.001**
**Day of illness**				
≥ 5	370 (72.7%)	139 (27.3%)	Ref	
< 5	96 (55.2%)	78 (44.8%)	**2.16** (**1.51-3.09)**	**<0.001**
**Having organ impairment at DSS**				
No	446 (68.8%)	202 (31.2%)	Ref	
.Yes	20 (57.1%)	15 (42.9%)	0.6 (0.3-1.2)	0.191*

* Fisher’s Exact Test **: Anova

Summary statistics are n (%) or mean ± SD.

Ref: reference group; DSS: Dengue shock syndrome.

**DSS associated with other severe manifestations:** Among 737 DSS patients, the most severe phenotype was the association of DSS, hemorrhage and organ impairment together, with a frequency of 3,8% (28/737 patients). Older age was associated with the most severe phenotype (Table 6). We also found a significant difference when comparing mean age between DSS group and “DSS + organ impairment” group (27.51 vs 30.28 years, p = 0.039), between DSS group and “DSS + organ impairment + hemorrhage” group (27.51 vs 32.07, p = 0.012). Additionally, having underlying disease was also a risk for hemorrhage and organ impairment together (p < 0.001), especially for those with diabetes (p < 0.001).

**Table 6:** Severe dengue phenotypes stratified according to sex, age, BMI, and comorbidity among patients with dengue shock syndrome (n = 737).

	DSS alone (N = 630)	DSS + severe hemorrhage (N = 26)	DSS + organ impairment (N = 53)	DSS + organ impairment + severe hemorrhage (N = 28)	P
**Gender**					
Male	302 (87.3)	16 (4.6)	19 (5.5)	9 (2.6)	Ref
Female	328 (83.9)	10 (2.6)	34 (8.7)	19 (4.9)	0.056
**Age**	27.51 ± 9.11	28.38 ± 8.65	30.28 ± 10.8	32.07 ± 12.69	0.018*
**Age groups**					
16-20	179 (87.7)	7 (3.4)	12 (5.9)	6 (2.9)	0.054
21-40	387 (86)	17 (3.8)	29 (6.4)	17 (3.8)	0.052
> 40	64 (77.1)	2 (2.4)	12 (14.5)	5 (6)	Ref
**BMI**					
< 25	416 (86.1)	21 (4.3)	31 (6.4)	15 (3.1)	
≥ 25	214 (84.3)	5 (2)	22 (8.7)	13 (5.1)	0.128
**Comorbidities**					
No	488 (88.4)	15 (2.7)	37 (6.7)	12 (2.2)	Ref
Yes	142 (76.8)	11 (5.9)	16 (8.6)	16 (8.6)	**<0.001**
**Liver disease**					
No	529 (86.2)	22 (3.6)	43 (7)	20 (3.3)	Ref
Yes	101 (82.1)	4 (3.3)	10 (8.1)	8 (6.5)	0.354
**Hypertension**					
No	612 (85.6)	26 (3.6)	52 (7.3)	25 (3.5)	Ref
Yes	18 (81.8)	0 (0)	1 (4.5)	3 (13.6)	0.077
**Diabetes**					
No	621 (86.5)	24 (3.3)	48 (6.7)	25 (3.5)	Ref
Yes	9 (47.4)	2 (10.5)	5 (26.3)	3 (15.8)	**<0.001**
**History of COVID-19 infection**					
No	552 (85.3)	23 (3.6)	45 (7)	27 (4.2)	Ref
Yes	78 (86.7)	3 (3.3)	8 (8.9)	1 (1.1)	0.496

*With posthoc ANOVA test, comparing age between DSS group vs DSS + organ impairment group (p = 0.039); between DSS group vs DSS + organ impairment + hemorrhage group (p = 0.012).

Summary statistics are n (%) or mean ± SD.

Ref: reference group; DSS: Dengue shock syndrome.

### Outcomes (Table 1)

At HTD, admission to the Intensive Care Unit (ICU) was indicated to DSS patients who did not respond to initial fluids and developed episodes of recurrent shock, patients who needed blood product transfusions, and who needed intensive interventions (therapeutic plasma exchange, mechanical ventilation).

The frequency of admission to ICU was 22.3% (199 patients), with a median stay at ICU only for 2 days (IQR: 2; 4 days), 67/891 patients (7.5%) had complications due to hospital-acquired infections (sepsis, pneumonia, urinary tract infection) with a longer hospital stay (the longest 25 days). 874 patients (98.1%) recovered fully. There were 11 fatal cases (1.2%), of which the median age was 26 (22; 43) years old, with the youngest 19 and the oldest 56 years old. 7 patients had BMI ≥ 25 and 4 had underlying diseases. Documented causes of death were: cerebral hemorrhage (2 cases within the first 3 days of hospitalisation), 9 cases due to septic shock and multi-organ failure (occurring during 18–25 days of hospitalisation).

## Discussion

Even though the clinical features of severe dengue were mentioned by many authors, there are limited descriptions of the complex clinical progression of adults with severe dengue in the literature. Our study provides the key picture of these cases, admitted and treated according to the standard Vietnamese Ministry of Health protocol, which is based on the WHO guidelines. For previous publications, the term was used as Severe dengue categories [6] while we define these as phenotypes, associated phenotypes for the same meaning: these severe manifestations occur simultaneously or overlap.

Our study population included all those ≥ 16 years old, but most were young adults (86.1% under 40 years old). Some authors [10] have reported more severe disease in elderly populations, but our study included too few older patients to draw any conclusions (only 8 patients > 60 years old). About gender, previous authors [6] have reported that severe disease in women was manifested as hemorrhage and organ involvement, however, this was not seen in our study.

Given the increasing prevalence of obesity in Vietnam in recent years [11], it is concerning that patients with greater BMI in our study experienced more episodes of recurrent shock. The association between obesity and overweight and dengue severity, especially with DSS, has already been documented in children [12] and our data show similar findings in adults. We also note that 5 of the 11 fatal cases were overweight patients and 2/11 cases were obese. We found that patients with BMI ≥ 25 had a higher prevalence of DSS accompanied by organ impairment, as well as DSS with both organ impairment and severe hemorrhage, compared to those with DSS alone (8.7% vs 6.4%; 5.1% vs 3.1%, p = 0.128) (Table 6). Our study was not conceived to examine whether overall patients who are overweight or obese are at greater risk of dengue or severe disease, but our findings support the hypothesis that severe disease is linked to BMI.

We also raise the question of why patients who had a COVID-19 infection in the past presented more episodes of recurrent shock than those who didn’t? Long COVID-19 is a multisystemic illness encompassing myalgic encephalomyelitis (chronic fatigue syndrome), dysautonomia, impacts on multiple organ systems, and vascular and clotting abnormalities [13]. It was demonstrated that SARS-CoV-2 affects both microvasculature and macrovasculature, causing both short and long-term vascular damage in the long-term [14]. Do these phenomena lead to more plasma leakage when the individual is infected with dengue who has a history of COVID-19? This hypothesis warrants further consideration, but assessing vascular damage directly was challenging.

Our data have shown the various phenotypes of severe dengue at first presentation, and the evolution of these cases during hospitalization. In line with the important mechanism of plasma leakage in dengue disease, the clinical shock phenotype was the commonest presenting feature, which is similar to severe dengue in children [6]. The association of recurrent shock with younger age supports the phenomenon of predominance of vascular leak in younger individuals [15]. On the other hand, a higher mean age was seen in patients who experienced associated severe manifestations as hemorrhage and organ impairment. The reasons for the differences between these 2 entities are not understood; it might be due to differences in the immune response of age groups or differences in susceptibility to other manifestations linked to comorbidity or other physiological factors.

Regarding preexisting underlying disease which may be related to clinical phenotypes of dengue patients, the presence of a higher frequency of diabetes patients in the most severe associated phenotypes was reported. In the 2009 WHO dengue guidelines [4], diabetes is noted as one of the risk factors for progression to severe illness. Through a review and meta-analysis [16], diabetes was associated with an increased risk for a severe clinical presentation of dengue (OR: 1.75; 95% CI: 1.08-2.84; p = 0.022). The same trend as our finding was found similarly in other research [17,18]. Similar to obesity, the rising prevalence of diabetes in Vietnamese people [19] is of concern for such hyperendemic areas.

Organ impairment and severe hemorrhage without shock reported at a frequency of 30% in our study were also described by other authors [20]. Cases of organ impairment were at a lower frequency compared to DSS, but they were hard to manage properly: 8/11 fatal cases experienced many organ failures without improvement with treatment. Our study detected 36/891 patients (4.0%) manifesting their organ involvement early in the illness course (before day 3 of illness). Clinicians should be aware of this risk, especially in patients with impaired consciousness or convulsions.

In our study, only 475 out of 891 patients (53.3%) tested positive for NS1 or IgM Dengue. This relatively low percentage may be attributable to the fact that many patients displayed severe manifestations on illness days 5–6 when the sensitivity of the NS1 dengue test is already reduced. It is possible that a small number of patients may not have had dengue? However, considering the clinicians’ extensive experience in treating dengue over a long period of time, together with the very classical clinical presentations, we believe that the number of patients who truly did not have dengue in this population is negligible. When comparing between the 2 groups of the study population (the group having lab confirmation and the group not having lab confirmation) to see whether this variable affects the outcomes: a) survival/death, b) with recurrent shock or without recurrent shock in DSS patients, c) presenting DSS alone or DSS with other severe manifestations. No significant differences were observed between the two groups (S1 Table: Comparison of outcomes between the 2 groups with and without having lab confirmation)

As a retrospective analysis, our study faced limitations due to the absence of direct patient reports regarding their dengue history, medical background, or comorbid conditions. Notably, our outcome data relied on hospital coding, preventing us from conducting any prospective assessments of quality-of-life metrics. We were unable to ascertain the prevalence of dengue serotypes during this outbreak or determine their immunological status (whether primary or secondary infections), which are known to influence disease severity [21]. These tests were not deemed essential for effective dengue management in Vietnam. Additionally, we could not execute a systematic review of laboratory tests, as these were conducted based on clinical indications.

For predicting severe progression, we based our analysis on demographic characteristics alone and did not include more complex features, laboratory values or predictive biomarkers, which have been shown to increase the performance of models [22]. Nevertheless, our results may guide the rational choice of biomarkers for future research or prognostication and are of practical value, in most Low and Middle-Income Countries where dengue occurs.

## Conclusion

We have provided a comprehensive description of the clinical phenotypes of severe dengue in Vietnamese adults during a major outbreak in Vietnam. This data describes the various manifestations and outcomes of adults affected by the disease. We confirm that dengue shock syndrome remains the most common feature, either alone or associated with severe hemorrhage or organ impairment. With the first approach based mainly on fundamental attributes (age, overweight and obesity status, comorbidities, day of illness, COVID-19 history) we found important to assess infection severity by a closely monitoring these at-risk adult patients in term of detecting early signs of recurrent shock among DSS patients, early signs of deterioration into organs failure, especially in health settings with limited laboratory resources.

## Supporting information

S1 Appendix Definition of severe dengue phenotypes. (DOCX)

S1 Table Comparison of outcomes between the 2 groups with and without having lab confirmation. (DOCX)

S1 Data Dataset. Data severe dengue.
(XLSX)
