# Supplementary material for: Severe dengue in adults: Clinical features from the 2022 dengue outbreak at a Vietnamese tertiary hospital
Source: PLoS Negl Trop Dis. 2025 Oct 3;19(10):e0013589. doi: 10.1371/journal.pntd.0013589 (PMC12513615; doi:10.1371/journal.pntd.0013589)
Supplement: S1 Appendix — (DOCX) [file pntd.0013589.s001.docx]

**S1 APPENDIX.** **Definition of severe dengue phenotypes**

|  | **Definition** |
| --- | --- |
| Dengue shock syndrome (DSS) | Shock attributable to plasma leakage. Patient is considered to have shock if the pulse pressure (difference between the systolic and diastolic pressures) is ≤ 20 mmHg. Shock is defined as severe if blood pressure and pulse are not measurable clinically, or prolonged shock if the circulatory compromise does not improve with the initial resuscitation within 6 hours. |
| Recurrent shock | Recurrent shock due to continuous plasma leakage after being hemodynamically stable for more than 6 hours post-resuscitation therapy. |
| Severe hemorrhage | Intensive bleeding needed any of intervention in the purpose of hemostasis such as[1]:   - Nasal package insertion, bandage compression - Gastroscopy for upper gastrointestinal hemostasis or using continuous intravenous proton pump inhibitor infusion - Bleeding at any body site needed blood products transfusion (platelets, fresh-packed red cells, cryoprecipitate) |
| **Severe organ involvement** | |
| Hepatic impairment | Liver injury is based only on the level of AST/ALT > 1000 U/L[2], without or with acute liver failure, demonstrated by INR ≥ 1.5 or hepatic encephalopathy[3-5]. |
| Renal impairment | Acute Kidney Injury (AKI) is defined as having any of the KDIGO 2012 criteria[6], and becomes severe if it progresses into stage 3 of kidney injury. |
| Cardiac impairment | Characterized by manifestations of cardiomyopathy[7] such as: acute myocardiac[8] or heart failure[9] (chest pain, dyspnea, tachycardia, shock, elevated cardiac enzymes and abnormalities in ECG or imaging)[2]. |
| Neurologic impairment | Changes in mental status: impaired consciousness, having convulsions or focal neurological signs with no other cause explaining the neurological manifestations[2]. |

# **Reference**

1. Vietnam Ministry of Health. Guidelines for diagnosis and treatment of dengue fever. 2023.

2. World Health Organization. Dengue guidelines for diagnosis, treatment, prevention and control : new edition. Geneva: World Health Organization; 2009.

3. Polson J., Lee W. M. AASLD position paper: the management of acute liver failure. Hepatology (Baltimore, Md). 2005;41(5):1179-97. Epub 2005/04/21. doi: 10.1002/hep.20703. PubMed PMID: 15841455.

4. Wendon Julia, Cordoba Juan, Dhawan Anil, Larsen Fin Stolze, Manns Michael, Nevens Frederik, et al. EASL Clinical Practical Guidelines on the management of acute (fulminant) liver failure. Journal of Hepatology. 2017;66(5):1047-81. doi: 10.1016/j.jhep.2016.12.003.

5. Vilstrup H., Amodio P., Bajaj J., Cordoba J., Ferenci P., Mullen K. D., et al. Hepatic encephalopathy in chronic liver disease: 2014 Practice Guideline by the American Association for the Study of Liver Diseases and the European Association for the Study of the Liver. Hepatology (Baltimore, Md). 2014;60(2):715-35. Epub 2014/07/22. doi: 10.1002/hep.27210. PubMed PMID: 25042402.

6. Khwaja A. KDIGO clinical practice guidelines for acute kidney injury. Nephron Clinical practice. 2012;120(4):c179-84. Epub 2012/08/15. doi: 10.1159/000339789. PubMed PMID: 22890468.

7. Richardson P., McKenna W., Bristow M., Maisch B., Mautner B., O'Connell J., et al. Report of the 1995 World Health Organization/International Society and Federation of Cardiology Task Force on the Definition and Classification of cardiomyopathies. Circulation. 1996;93(5):841-2. Epub 1996/03/01. doi: 10.1161/01.cir.93.5.841. PubMed PMID: 8598070.

8. Ammirati Enrico, Frigerio Maria, Adler Eric D., Basso Cristina, Birnie David H., Brambatti Michela, et al. Management of Acute Myocarditis and Chronic Inflammatory Cardiomyopathy. 2020;13(11):e007405. doi: doi:10.1161/CIRCHEARTFAILURE.120.007405.

9. Members: Authors/Task Force, McDonagh Theresa A., Metra Marco, Adamo Marianna, Gardner Roy S., Baumbach Andreas, et al. 2021 ESC Guidelines for the diagnosis and treatment of acute and chronic heart failure. 2022;24(1):4-131. doi: <https://doi.org/10.1002/ejhf.2333>.
