# Supplementary material for: Severe dengue in adults: Clinical features from the 2022 dengue outbreak at a Vietnamese tertiary hospital
Source: PLoS Negl Trop Dis. 2025 Oct 3;19(10):e0013589. doi: 10.1371/journal.pntd.0013589 (PMC12513615; doi:10.1371/journal.pntd.0013589)
Supplement: S1 Table — (DOCX) [file pntd.0013589.s002.docx]

**S1 Table. Comparison of outcomes between the 2 groups with and without having lab confirmation**

|  | **Survived**  **(n=880)** | **Death**  **(n=11)** | **P value** |
| --- | --- | --- | --- |
| No test NS1/IgM dengue positive | 412 (99) | 4 (1) | Ref |
| NS1/IgM dengue positive | 468 (98.5) | 7 (1.5) | 0.353* |

* Fisher’s Exact; Ref: Reference group; N = 891 patients with severe dengue

|  | **DSS without recurrent shock**  **(n = 487)** | **DSS with recurrent shock (n = 250)** | **P value** |
| --- | --- | --- | --- |
| No test NS1/IgM dengue positive | 247 (67.5) | 119 (32.5) | Ref |
| NS1/IgM dengue positive | 240 (64.7) | 131 (35.3) | 0.423 |

Chi-squared test; Ref: Reference group; DSS: dengue shock syndrome; N = 737 patients with dengue shock syndrome

|  | DSS alone  (n=630) | DSS + severe hemorrhage  (n=26) | DSS + organ impairment  (n=53) | DSS + organ impairment + severe hemorrhage  (n=28) | P value |
| --- | --- | --- | --- | --- | --- |
| No test NS1/IgM dengue positive | 321 (87.7) | 14 (3.8) | 21 (5.7) | 10 (2.7) | Ref |
| NS1/IgM dengue positive | 309 (83.3) | 12 (3.2) | 32 (8.6) | 18 (4.9) | 0.178 |

Chi-squared test; Ref: Reference group; DSS: dengue shock syndrome; N = 737 patients with dengue shock syndrome
